# MARIS: Method for Analyzing RNA following Intracellular Sorting

**DOI:** 10.1371/journal.pone.0089459

**Published:** 2014-03-03

**Authors:** Siniša Hrvatin, Francis Deng, Charles W. O'Donnell, David K. Gifford, Douglas A. Melton

**Affiliations:** 1 The Harvard Department of Stem Cell and Regenerative Biology, Harvard Stem Cell Institute, Harvard University, Cambridge, Massachusetts, United States of America; 2 Computer Science and Artificial Intelligence Laboratory, Massachusetts Institute of Technology, Cambridge, Massachusetts, United States of America; 3 Howard Hughes Medical Institute, Harvard University, Cambridge, Massachusetts, United States of America; University of Tampere, Finland

## Abstract

Transcriptional profiling is a key technique in the study of cell biology that is limited by the availability of reagents to uniquely identify specific cell types and isolate high quality RNA from them. We report a Method for Analyzing RNA following Intracellular Sorting (MARIS) that generates high quality RNA for transcriptome profiling following cellular fixation, intracellular immunofluorescent staining and FACS. MARIS can therefore be used to isolate high quality RNA from many otherwise inaccessible cell types simply based on immunofluorescent tagging of unique intracellular proteins. As proof of principle, we isolate RNA from sorted human embryonic stem cell-derived insulin-expressing cells as well as adult human β cells. MARIS is a basic molecular biology technique that could be used across several biological disciplines.

## Introduction

New technologies including microarrays and RNA-seq have greatly advanced our understanding of cells and cell states. The potential of these approaches, however, has been limited by the ability to isolate and purify specific cell types of interest from complex cellular mixtures and tissues, and then analyze their transcriptional profile. Antibodies to cell surface markers and genetic reporter lines allow access only to a few distinct cell types in model organisms and are even more limiting in the study of human cells. Most cell types can be identified and isolated based on the expression of intracellular markers, but the process of intracellular immunofluorescent labeling is generally thought to degrade the RNA in the cells, compromising accurate downstream gene expression analysis. The ability to isolate and accurately transcriptionally profile cells based on intracellular antibody staining could allow us to analyze gene expression in almost any cell or tissue.

We sought to develop new tools to isolate high-quality RNA from cells following intracellular antibody staining and fluorescence-activated cell sorting (FACS). Previously, RNA of sufficient quality for FISH, nuclease protection assays, RT-PCR and microarray analysis has been obtained following fixation, intracellular immunofluorescent staining, and FACS or laser capture microdissection (LCM) [1]–[8]. However, these publications do not rigorously address whether these relatively harsh manipulations produce biased results at the transcriptome level due to crosslinking and partial degradation of RNA.

We developed a Method for Analyzing RNA following Intracellular Sorting (MARIS) that generates RNA of high quality for transcriptome profiling, including microarray analysis and RNA-seq, following cellular fixation, intracellular immunofluorescent staining and FACS. Using MARIS, we isolated high quality RNA from heterogeneous cultures of differentiated human embryonic stem cells (hESCs) as wells as primary human pancreatic tissue. Broadly speaking, MARIS may be used for the transcriptional characterization of cells solely based on immunofluorescent detection of intracellular proteins in the absence of reporter lines or sortable cell surface markers.

Directed differentiation of hESCs has the potential to produce virtually unlimited quantities of any cell type for cell transplantation therapy. Stepwise directed differentiation protocols have been used to produce hESC-derived insulin-expressing cells (hESC-INS^+^) cells. However, the degree to which these hESC-INS^+^ cells resemble adult human insulin-expressing β cells remains unclear due to the lack of tools for the isolation of either pure cell type. Here we present an application of MARIS for the isolation of high quality RNA from hESC-INS^+^ cells and sorted adult human β cells.

## Results

### RNA isolation from fixed, stained and sorted cells

**Figure 1:**
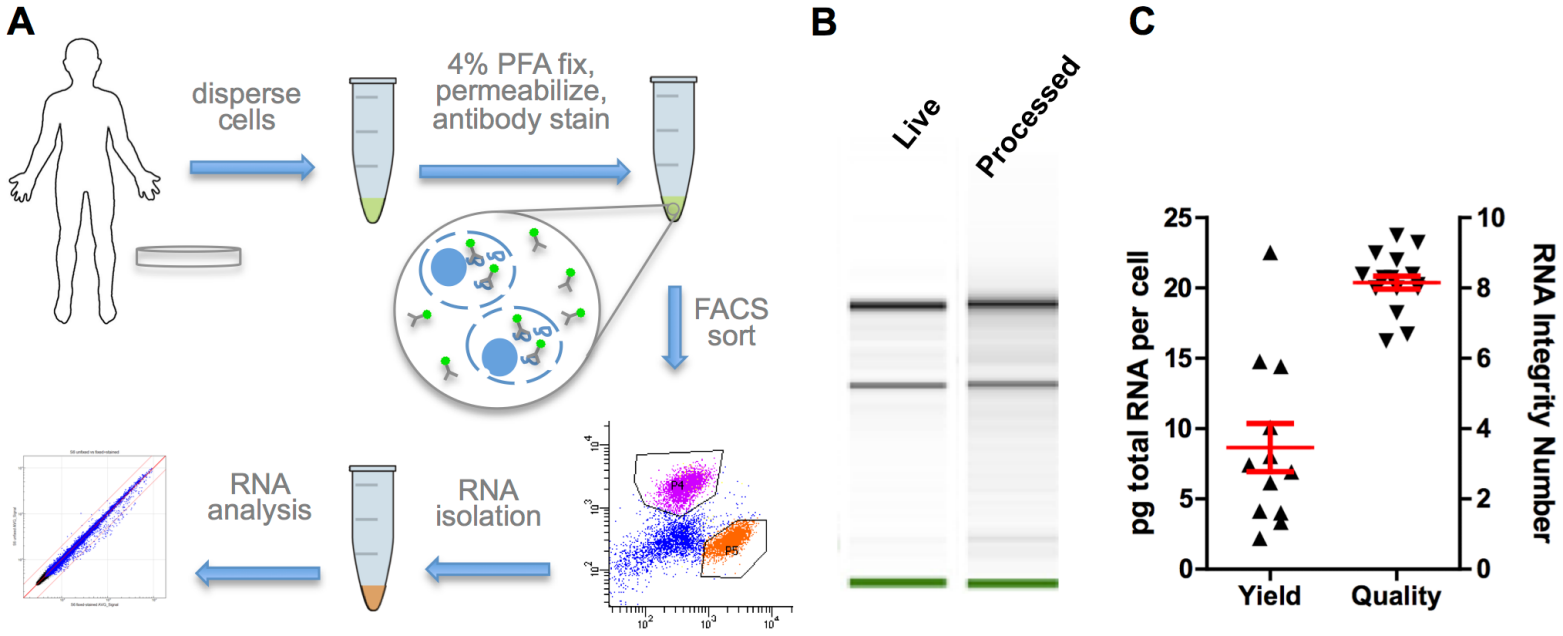
High quality RNA isolation from fixed and stained cells. (A) Outline of the developed protocol. *In vivo* or *in vitro*-derived cells are dispersed, fixed in 4% PFA, permeabilized, stained using standard immunofluorescent antibodies and FACS sorted. Total RNA is isolated using a modified RNA extraction protocol (see methods). (B) RNA was isolated and analyzed from hESC-derived Stage 6 cells before fixation (live) or following fixation, staining and sorting (processed). Simulated electropherogram suggests minimal degradation of total RNA based on the clearly defined 18S and 28S ribosomal RNA bands; RIN value 8.1 for live, 8.0 for processed sample. (C) RNA was isolated from multiple samples across three independent experiments. The average RIN score was 8.3±0.7 (mean±SEM, n = 14) and the average yield 8.35±1.61 pg total RNA per cell (mean±SEM, n = 13).

We combined, modified, and optimized existing protocols and kits to generate a protocol that extracts high quality RNA from fixed cells that have been sorted based on intracellular immunofluorescence (Fig. 1a, Materials and Methods). hESC-lines H1 [9] and HUES8 [10], differentiated to the final stage of our pancreatic differentiation protocol (modified from [11]) were used as starting material (Stage 6, Fig. S1A). Several assays were used to compare the quality of the RNA isolated using this protocol and RNA isolated from live (fresh, unfixed) cells. RNA was extracted from cells following fixation, permeabilization, antibody staining, and FACS whereby all the cells were collected (processed cells). Control RNA was extracted from live unsorted cells using the Qiagen RNAeasy kit. In parallel preparations, isolated RNA demonstrated RNA Integrity Numbers (RINs) of 8.1 (live) and 8.0 (processed, Fig. 1b). The RNA quality was highly reproducible across independent preparations and different cell types with average RIN score of 8.3±0.7 (n = 14 samples, Fig. 1c). MARIS yielded 8.35±1.61 pg total RNA per cell (n = 13 samples; Fig. 1c), which is within the normal limits for human cells [12].

### Transcriptional bias analysis

**Figure 2:**
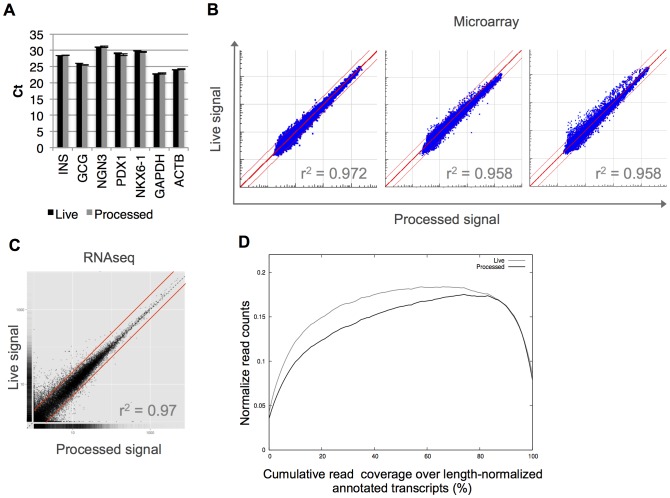
Quantitative comparison of live and processed cells. (A) qRT-PCR on live and processed Stage 6 cells (n = 3) for pancreatic and housekeeping genes. (B) Logarithmic scatter plots of Illumina microarray data between live and processed stage 6 samples show r^2^ = 0.963±0.005 (mean±SEM, n = 3, r^2^ determined by Pearson's correlation) correlation for all detected probes (detection p<0.05). Red lines represent 2-fold change. (C) Samples were prepared and paired-end sequenced using TruSeq chemistry on a HiSeq 2000 (Illumina). GENCODE per-gene FPKM values on a logarithmic plot. r^2^ = 0.97 (Pearson's correlation). Red lines represent 2-fold change. (D) Relative RNA-seq coverage of all annotated transcripts shows 3′ bias in longer length genes. Live and processed RNA-seq read coverage over length-normalized GENCODE transcripts (Live area under the curve 0.133, Processed area under the curve 0.123). Coverage counts were normalized by per-experiment sequencing depth.

Having confirmed the integrity of RNA isolated using MARIS, we next assessed whether the protocol changed the representation of individual transcripts (in case the MARIS procedure selectively depleted or enriched for some RNA species). We first performed qRT-PCR analysis of RNA extracted from live and processed Stage 6 cells for several housekeeping genes, as well as genes specific to the pancreatic lineage (Fig. 2a). There was no systemic statistically significant (paired two-tailed t-test) difference in cycle threshold values between live and processed cells.

To evaluate the impact of MARIS at the whole-genome level, RNA from live and processed cells was analyzed using Illumina microarrays (Fig. 2b). Across all detected genes, expression between live and processed samples was very similar (r^2^ = 0.963±0.008, n = 3). In each pair, the number of genes differentially expressed between the live and processed sample (fold change of 2) was 41±18. No genes were consistently differentially expressed between live and processed cells suggesting that MARIS does not introduce systemic changes in gene expression. Finally, analysis by RNA-seq showed very similar gene expression between live and processed cells (r^2^ = 0.97, Fig. 2c). Degradation of RNA results in increased detection of transcripts at the 3′ end relative to the 5′ end (3′-bias) [13]. Processing increased to a small degree RNA-seq transcript 3′-coverage bias (Fig. 2d). Together, these analyses confirm that MARIS produces high-quality RNA and has little effect on the representation of transcripts as analyzed at the level of the individual gene, or through two methods of genome-wide analysis.

### RNA isolation from insulin expressing cells generated from hESCs and adult human islets

**Figure 3:**
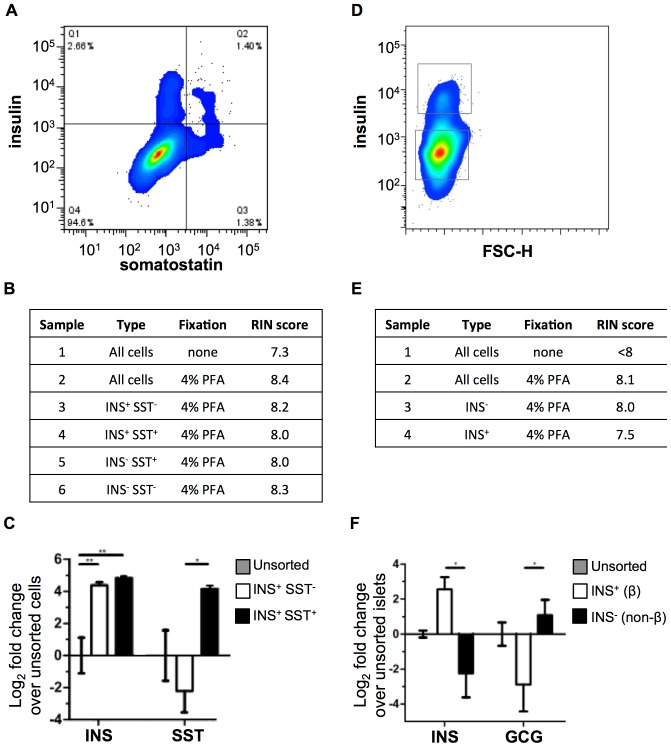
Sorting of insulin-expressing cells from human pluripotent stem cells and adult human islets. (A) FACS plot of Stage 6 H1-derived cells sorted for insulin and somatostatin. (B) RNA samples were isolated from sort in panel A. RIN scores indicate RNA quality. (C) qRT-PCR of unsorted cells compared to and INS^+^ SST^−^ cells and INS^+^ SST^+^ cells (Paired two-tailed t-test; * p<0.05, **p<0.01). (D) FACS plot of human adult islets sorted for insulin (INS). (B) RNA samples were isolated from sort in panel D. RIN scores indicate RNA quality. (C) qRT-PCR of unsorted islets compared to and INS^+^ and INS^−^ cells (Paired two-tailed t-test; * p<0.05).

hESCs were differentiated to Stage 6 cells, fixed, stained for insulin and somatostatin, and sorted for RNA isolation. INS^+^ cells comprised approximately 4% of all Stage 6 cells (Fig. 3a). A large proportion of INS^+^ cells also co-expressed the pancreatic hormone somatostatin, consistent with previous reports [11], [14]. RIN values for RNA isolated from sorted stage 6 cells were ≥8 (Fig. 3b, Fig. S2a). qRT-PCR for insulin and somatostatin indicated significantly enriched expression of these endocrine hormones in the sorted populations, confirming successful purification of INS^+^ SST^−^ and INS^+^ SST^+^ cells (Fig. 3c).

Next, we isolated adult human β cells from an islet preparation of a post-mortem donated human pancreas. About 10% of the cells in the preparation were insulin-expressing human β cells (Fig. 3d). RIN values for RNA isolated from sorted adult β cells were ≥7.5 (Fig. 3e, Fig. S2a). qRT-PCR for insulin and glucagon indicated significant enrichment of insulin in the INS^+^ population and of glucagon in the INS^−^ population, indicating successful purification of human insulin-expressing β cells from other islet cells (Fig. 3f).

## Discussion

Most cell types cannot be isolated due to the absence of specific cell surface markers and dyes to uniquely identify them. Instead, these cells can be identified and isolated based on the expression of intracellular markers. Intracellular staining, however, requires permeabilization, chemical fixation, and use of reagents that degrade RNA, hindering downstream gene expression analysis.

This study reports a Method for Analyzing RNA following Intracellular Sorting (MARIS) for obtaining high quality RNA suitable for transcriptome profiling following cellular fixation, intracellular immunofluorescent staining and FACS. The results show that MARIS routinely produces RNA with RIN scores above 8 that can be used for qRT-PCR, microarray and RNA-seq analysis without detectable loss of fidelity.

MARIS permits analysis of genetically unmodified cells produced by differentiating hESCs *in vitro*, and the direct comparison of these cells to corresponding cell types isolated from human tissues. Using MARIS we've isolated RNA from hESC-INS^+^ cells, as well as sorted human β cells. We intend to characterize and compare the gene expression of these two cell populations to determine their degree of similarity and identify candidate genes that would help us generate hESC-INS^+^ cells that more closely resemble adult human β cells.

In addition to the use of hormones such as insulin and somatostatin for intracellular FACS, MARIS has been successfully used with antibodies against transcription factors such as PDX1 and NKX6-1 (data not shown). Each antibody will require optimization of antibody concentration, and the length of incubation time during the primary antibody stain.

Improvements to RNA-Seq technology allow for the sequencing of single cells [15]. To expand the use of MARIS, it would be interesting to test whether it is compatible with single cell RNA-Seq protocols.

In summary, MARIS is tool of broad utility across biological disciplines to analyze gene expression of previously inaccessible cell types based on intracellular markers. We anticipate that it will be of particular use in the study of human biology.

## Experimental Procedures

### MARIS Staining and FACS

hPSC-derived cells and human islets were dispersed to a single cell suspension using TrypLE Express (Invitrogen). Cells were passed through a 40 µm filter (BD Falcon 352340) and washed with PBS at least twice. Cells were fixed and permeabilized with 4% PFA (Electron Microscopy Sciences), 0.1% saponin (Sigma-Aldrich 47036) in molecular grade PBS (Ambion) supplemented with 1∶100 RNasin Plus RNase Inhibitor (Promega, N2615) for 30′ at 4**°**C. All the subsequent steps were carried out at 4**°**C. Cells were pelleted by centrifugation at 3000g for 3′ at 4**°**C and washed in Wash Buffer: PBS containing 0.2% BSA (Gemini Bio-Products), 0.1% saponin (Sigma-Aldrich), 1∶100 RNasin Plus RNase Inihibitor. Primary antibody staining was carried out while 3D rocking for 30′ at 4**°**C in Staining buffer containing PBS with 1% BSA, 0.1% saponin and 1∶25 RNasin Plus RNase Inhibitor (note that certain antibodies may require longer incubation times). Cells were washed twice in Wash Buffer followed by secondary antibody staining in Staining buffer. Following secondary antibody staining cells were washed twice in Wash buffer and resuspended in Sort buffer containing PBS, 0.5% BSA, and 1∶25 RNasin Plus RNase Inhibitor. Fixation, washing, staining and sorting were performed at a concentration of 5–10 M cells/ml. The list of primary and secondary antibodies used is provided in Table S1.

Cells were sorted on the FACSAria (BD Biosciences) using FACSDiva software. Gates were set with reference to negative controls. The sorting speed was adjusted to ensure sorting efficiency above 90%. Cells were collected in tubes that were coated with a small amount of Sort buffer.

### RNA isolation

After sorting, cells were pelleted by centrifugation at 3000 g for 5′ at 4°C. The supernatant was discarded. Total RNA was isolated from the pellet using the RecoverAll Total Nucleic Acid Isolation kit (Ambion), starting at the protease digestion stage of manufacturer-recommended protocol. The following modification to the isolation procedure was made: instead of incubating cells in digestion buffer for 15 minutes at 50°C and 15 minutes at 80°C, we carried out the incubation for 3 hours at 50°C. Cell lysates were frozen at −80°C overnight before continuing the RNA isolation by the manufacturer's instructions.

### Quantitative RT-PCR

Complementary DNA (cDNA) was generated from 4 ng of total RNA with random hexamer priming using the High Capacity cDNA Reverse Transcription with RNase Inhibitor kit (Applied Biosystems). One-fourth of the cDNA was used for each TaqMan qRT-PCR reaction using the Fast Universal PCR Master Mix with no AmpErase UNG (Applied Biosystems). The list of used probes is provided in Table S2. Reactions were run on an Applied Biosystems 7900HT Fast Real-Time PCR System with default settings. Detection thresholds were automatically computed by SDS 2.3 software (Applied Biosystems). Threshold data were analyzed in DataAssist 3.0 (Applied Biosystems) using the Comparative Ct (ΔΔCt) relative quantitation method, using β-actin as the endogenous control.

### Global gene expression analysis - microarray

Using the Illumina TotalPrep RNA Amplification kit (Ambion), double-stranded cDNA was generated following reverse transcription from 100 ng of total RNA. *In vitro* transcription overnight with biotin-labeled nucleotides created amplified mRNA (cRNA), which was concentrated by vacuum centrifugation at 30°C. 750 ng cRNA per sample was then hybridized to Human HT-12 Expression BeadChips (Illumina) using the Whole- Genome Expression Direct Hybridization kit (Illumina). Finally, chips were scanned on the Illumina Beadstation 500. The chip annotation manifest was version 4, revision 1. For differential expression analysis and the generation of gene lists for functional annotation and pathway analysis, microarray data were processed in GenomeStudio (Illumina, V2011.1). Raw data were adjusted by background subtraction and rank-invariant normalization. Before calculating fold change, an offset of 20 was added to all probe set means to eliminate negative signals. The p- values for differences between mean signals were calculated in GenomeStudio by t-test and corrected for multiple hypotheses testing by the Benjamini-Hochberg method in combination with the Illumina custom false discovery rate (FDR) model. Microarray data have been uploaded to GEO (accession number GSE54179).

### Global gene expression analysis – RNA-seq

Isolated RNA was obtained from 2 biological replicates of HUES8-derived INS^+^ cells and human adult β cell, as well as one replicate of live and processed stage 6 cells. Samples were poly-A purified and converted to cDNA libraries using the Illumina TruSeq protocol, and prepared into Illumina libraries using the Beckman Coulter Genomics SPRI-works system using custom adapters. 6 nt 3′ barcodes were added during PCR enrichment and the resulting fragments were evaluated using Agilent BioAnalyzer 2100. Samples were multiplexed 2-per-lane for sequencing using the Illumina HiSeq 2000 platform with paired-end read lengths of 80 nt, resulting in 68 M to 112 M paired reads per sample, and an average biological fragment length of 168–179 nt. Reads were aligned to the human genome (GRCh37/hg19) using STAR (version 2.2.0c) guided by GENCODE gene annotations (version 14) [16]. RNA-seq FPKM (fragments per kilobase of exon per million fragments) gene enrichment was determined using maximum likelihood by Cuffdiff [17], [18] (version 2.0.2), and visualized using CummeRbund [17]. Transcript differential expression was calculated by Cuffdiff using the default negative binomial model, with significant hits also confirmed using the count-based technique DESeq [19]. RNA-seq data have been uploaded to GEO (accession number GSE54179).

### Ethics Statement

Human pancreatic islets from non-diabetic donors were obtained through the National Disease Research Interchange (http://ndriresource.org/Human-Tissue-Services/Pancreatic-Islets/31/) with appropriate consent. All tissue samples were rendered anonymous. The use of human tissue was approved by the Harvard University Committee on the Use of Human Subjects in Research.

## Supporting Information

Figure S1 **Directed differentiation protocol.** Stepwise differentiation from hESCs to pancreatic endocrine cells. DE, definitive endoderm; PP, pancreatic progenitor; EP, endocrine progenitor; EN, endocrine cells. Table contains reagents used during each stage of directed differentiation.
(TIFF)

Figure S2 **RNA quality from sorted cells.** (A) Electropherograms of RNA from samples in Figure 3B, hESC-derived Stage 6 cells sorted for insulin and somatostatin. (B) Electropherograms of RNA from samples in Figure 3D, adult human islets sorted for insulin.
(TIFF)

Table S1 **Antibodies.** List of all antibodies used in the study.
(TIFF)

Table S2 **Taqman probes.** List of Taqman probes for qRT-PCR used in the study.
(TIFF)
