# Supplementary figures and images for: MARIS: Method for Analyzing RNA following Intracellular Sorting
Source: PLoS One. 2014 Mar 3;9(3):e89459. doi: 10.1371/journal.pone.0089459 (PMC3940959; doi:10.1371/journal.pone.0089459)

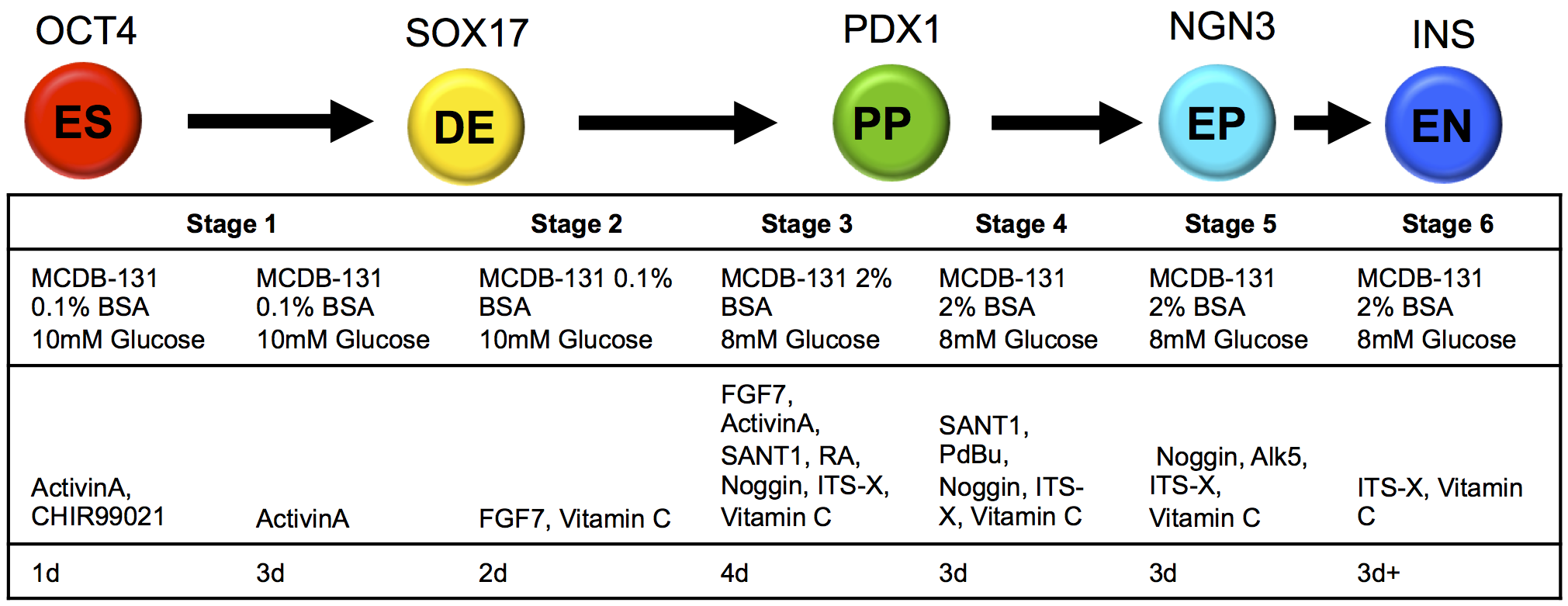

Supplement: Figure S1 — Directed differentiation protocol. Stepwise differentiation from hESCs to pancreatic endocrine cells. DE, definitive endoderm; PP, pancreatic progenitor; EP, endocrine progenitor; EN, endocrine cells. Table contains reagents used during each stage of directed differentiation. (TIFF) [file pone.0089459.s001.tiff]

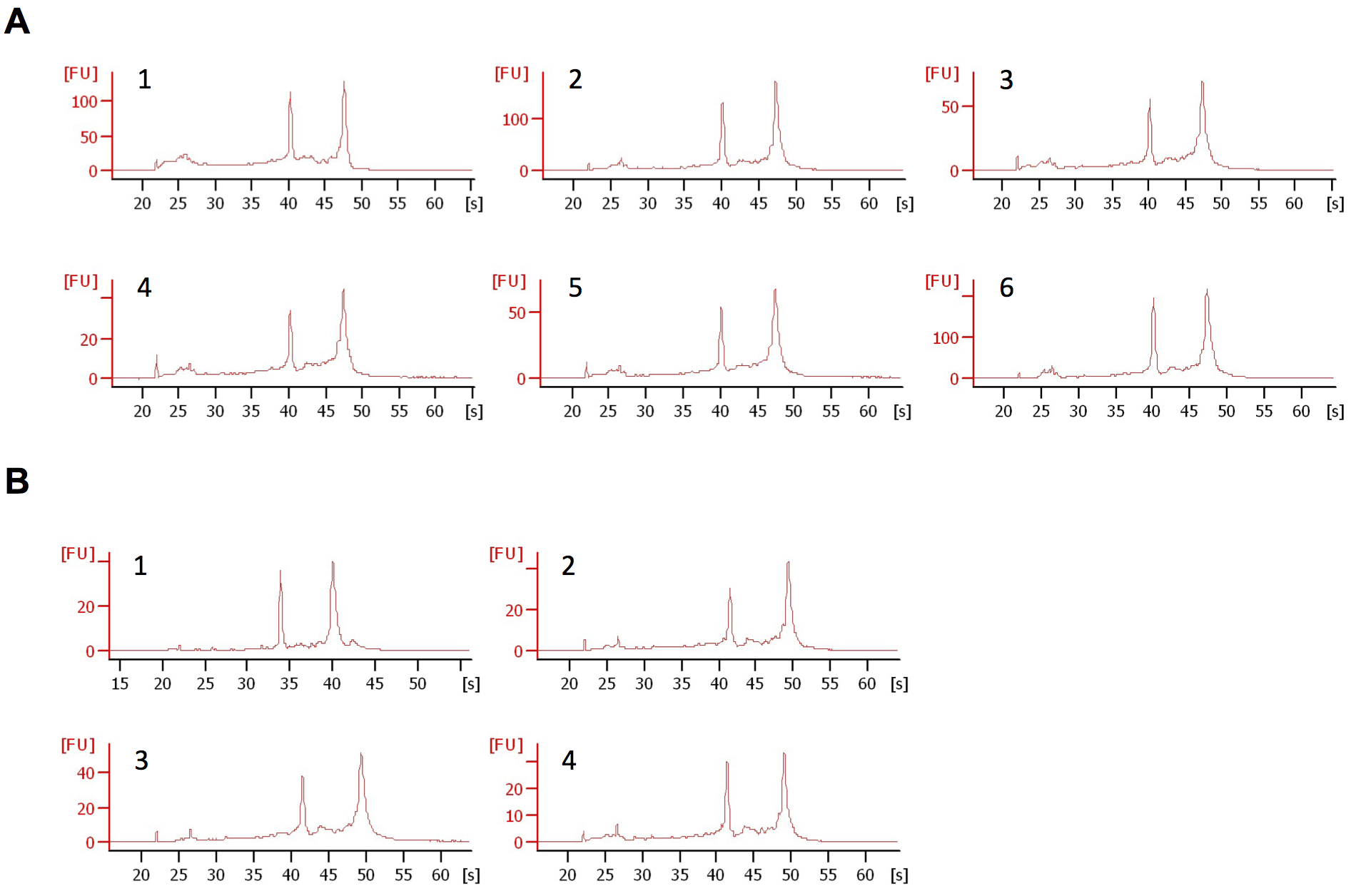

Supplement: Figure S2 — RNA quality from sorted cells. (A) Electropherograms of RNA from samples in Figure 3B, hESC-derived Stage 6 cells sorted for insulin and somatostatin. (B) Electropherograms of RNA from samples in Figure 3D, adult human islets sorted for insulin. (TIFF) [file pone.0089459.s002.tiff]

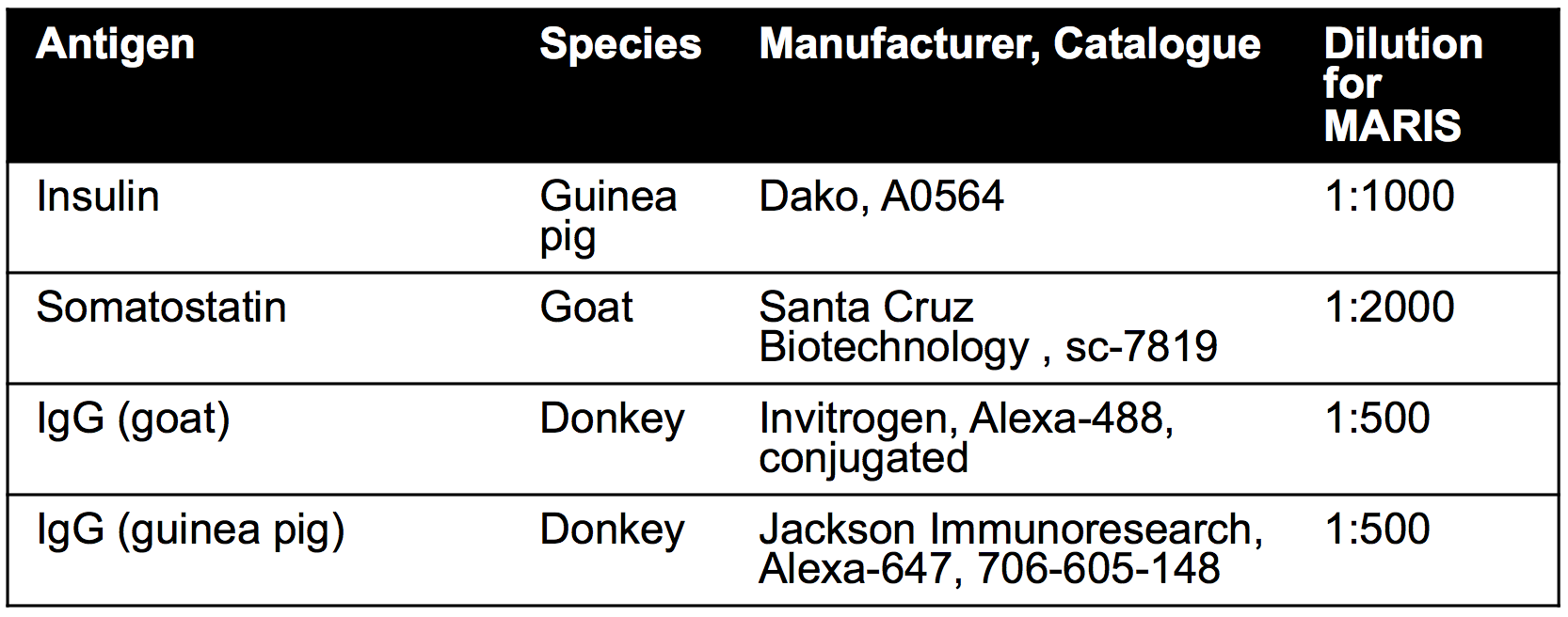

Supplement: Table S1 — Antibodies. List of all antibodies used in the study. (TIFF) [file pone.0089459.s003.tiff]

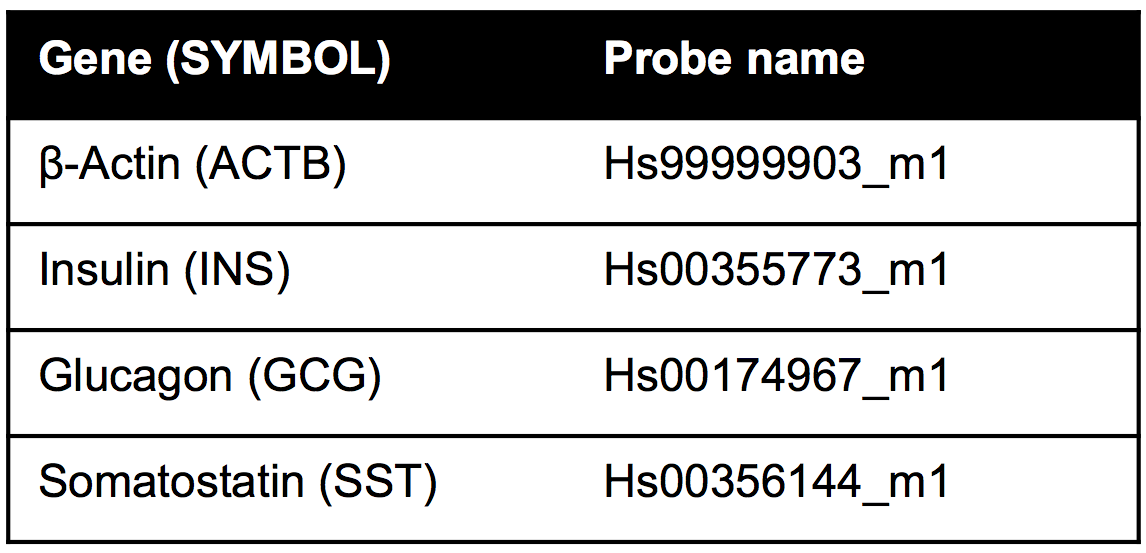

Supplement: Table S2 — Taqman probes. List of Taqman probes for qRT-PCR used in the study. (TIFF) [file pone.0089459.s004.tiff]
